# Non-spherical oscillations and induced microstreaming of single bubble in standing wave field

**DOI:** 10.1016/j.ultsonch.2026.108035

**Published:** 2026-09-02

**Authors:** Zhongyang Wu, Jinfu Liang, Chang Gao

**Affiliations:** School of Physics and Electronic Science, Guizhou Normal University, Guiyang 550025, China

**Keywords:** Standing wave field, Bubble non-spherical oscillation, Acoustic microstreaming

## Abstract

The transition of a freely suspended microbubble from spherical to nonspherical oscillation in a standing-wave acoustic field, together with the transient flow generated during this transition, was investigated using a combined experimental and numerical approach. In the experimental component, a single bubble was levitated near the center of the acoustic field using an ultrasonic levitation system. High-speed imaging captured the shape transition of the bubble from spherical to nonspherical oscillation and the trajectories of surrounding particles induced by the nonspherical bubble oscillation. These trajectories were subsequently superimposed to reconstruct the acoustic microstreaming structures. In the numerical component, simulations based on the Navier–Stokes equations and arbitrary Lagrangian–Eulerian (ALE) method with moving grids were performed to reproduce the transition from spherical to nonspherical oscillation and to resolve the transient vortex flow evolving throughout this transition. Furthermore, parametric studies revealed that driving frequency, bubble radius, and driving pressure significantly modulate the nonspherical oscillation modes and the resulting transient vortex topology. The mechanisms underlying vortex structure formation and the distribution characteristics of shear stress during multimode-coupled bubble oscillations were elucidated. These findings provide valuable insights for the application of acoustic microstreaming in particle manipulation, targeted drug delivery, and related fields.

## 1 Introduction

When ultrasonic waves propagate through a liquid, the bubble nuclei present within the liquid can expand into micrometer-sized bubbles visible to the naked eye. This phenomenon is referred to as acoustic cavitation [1]. The resulting bubbles are known as cavitation bubbles. The dynamic characteristics of cavitation bubbles are influenced not only by the ultrasonic frequency and pressure amplitude but also by the physical properties of the liquid, including density, viscosity, and bubble surface tension. Under specific ultrasonic field conditions, continuous excitation over multiple periods causes bubbles to transition from a spherical to nonspherical oscillation, resulting in various shape mode oscillations [2]. Purely spherical oscillations of an isolated bubble in an unbounded fluid do not generate any steady flow in the surrounding liquid [3]. By contrast, when a bubble is situated near a boundary or undergoes non-spherical oscillations, it induces a mean flow in the adjacent fluid, a phenomenon known as microstreaming [4]. Acoustic microstreaming technology demonstrates considerable potential for applications in micro-mixing [5], [6], targeted drug delivery [7], [8], particle manipulation [9], cell capture [10], and the separation of high-purity exosomes [11].

Early investigations primarily focused on acoustic microstreaming produced by bubbles near surfaces. Elder [4] and Kolb et al. [12] were the first to experimentally document the distinct flow patterns associated with bubble-induced acoustic microstreaming. Using particle image velocimetry (PIV), Tho et al. [13] examined the acoustic microstreaming generated by bubbles adhering to solid boundaries. Subsequently, Mobadersany [14] and Fauconnier et al. [15] expanded this research by investigating acoustic microstreaming generated by bubbles in contact with walls, thereby improving the understanding of near-wall acoustic microstreaming and facilitating its applications in ultrasonic cleaning and biological effects. However, the presence of walls can interfere with the flow field, making it difficult to experimentally obtain complete and accurate flow information around free bubbles. In recent years, Doinikov and Inserra et al. [16], [17], [18], [19] developed a theoretical framework for acoustic microstreaming induced by multimode-coupled oscillations of a single bubble in a free acoustic field. Inserra et al. [20] further decomposed the velocity field and provided an exact solution for the three-dimensional steady-state microstreaming field under asymmetric oscillations. Additionally, Guédra [21] examined the effect of driving ultrasonic pressure amplitude on the non-spherical oscillation modes of cavitation bubbles. Cleve et al. [22] were the first to employ acoustic levitation bubble-capture technology to investigate the non-spherical oscillation of bubbles in free space and the resulting acoustic microstreaming. Using a combination of experimental and simulation approaches, Mondal et al. [23] analyzed variations in liquid velocity when bubbles oscillated non-spherically near the pressure threshold. Although the studies have significantly advanced the understanding of microstreaming associated with fully developed, stable nonspherical oscillations, the dynamic transition of a bubble from spherical to nonspherical oscillation, as well as the onset and evolution of flow field structure remain relatively underexplored.

In this study, we experimentally capture and numerically simulate the dynamic transition of bubbles from spherical to nonspherical oscillation and the resulting acoustic microstreaming. A tunable standing-wave acoustic platform levitates a single free bubble via acoustic radiation force, driving its periodic oscillation at the acoustic frequency. A high-speed camera records the shape evolution during this transition, while tracer particles reveal the surrounding microstreaming patterns. Finally, finite-element simulations investigate the coupling between bubble surface deformation and the adjacent flow field. Our results reveal that during the spherical to nonspherical transition, the instantaneous oscillatory flow field reorganizes through a series of qualitatively distinct patterns, which in turn drive the progressive evolution of the time-averaged acoustic microstreaming toward its steady state. These findings provide a mechanistic basis for predicting and controlling microstreaming during shape-mode transitions, with potential implications for acoustic-based particle manipulation, targeted drug delivery, and related applications.

## 2 Experiment and simulation

### 2.1 Experimental setup and methods

**Fig. 1:**
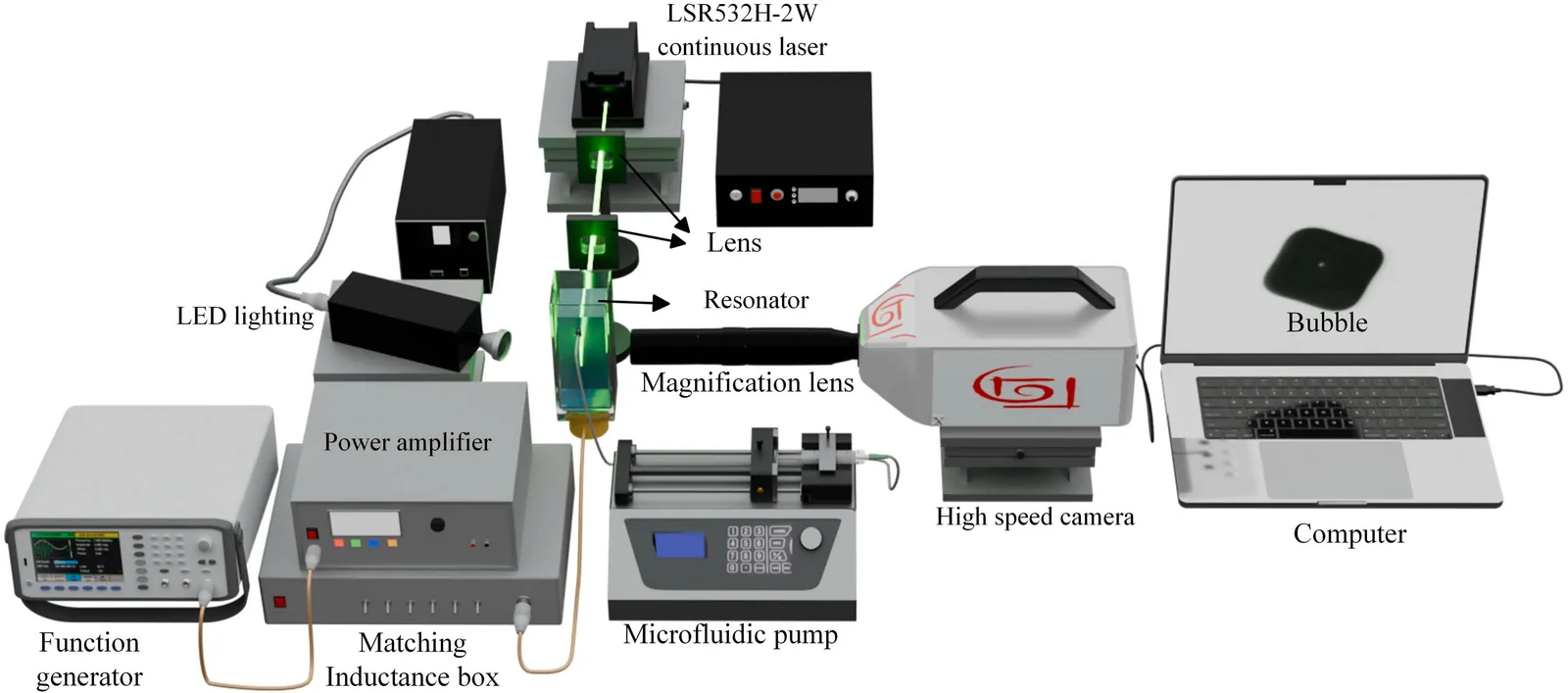
Three-dimensional experimental setup system diagram.

Fig. 1 illustrates the measurement device designed to assess the nonspherical oscillation behavior of bubbles and acoustic microstreaming. This system comprises three primary components: an ultrasonic drive system, high-speed camera acquisition control system, and lighting system. The ultrasonic drive system comprises a digital signal generator (Keysight 33600A), power amplifier (Aigtek ATA-2021H), matching inductance box, and an acoustic resonant cavity measuring 5 cm × 5 cm × 20 cm. This cavity is equipped with a pair of piezoelectric ceramic transducers (PZT, approximately 3 cm in diameter), and affixed to the bottom. In addition, the system includes a microfluidic pump. The high-speed acquisition control system features a Revealer high-frame-rate camera (Revealer X100), which is fitted with an optical magnification lens that provides 20-fold magnification. The lighting system consists of a Light Emitting Diode (LED) lighting system (HECHO S5000D) and a continuous laser lighting system (LSR532H-2W). The LED lighting system is utilized to capture the nonspherical multimode oscillations of a cavitation bubble, while the laser system was employed for imaging acoustic microstreaming. The continuous laser system comprises a continuous laser generator, double-sided concave lens, and plano-convex cylindrical lens forming plane light sheet.

In this experiment, the ultrasonic driving system was employed to generate non-spherical oscillations of cavitation bubbles and acoustic microstreaming. The signal generator produced a sinusoidal ultrasonic signal at a driving frequency of 24 kHz, corresponding to an acoustic wavelength of approximately 62.5 mm in water, which is far larger than the micrometer-scale bubbles. The signal was amplified by the power amplifier and matched with the inductive coil. It drove the PZT located at the bottom of the resonant cavity. The resonator contained 300 ml of deionized water with an oxygen content of 6.5 mg/L, which allowed the formation of a stable acoustic standing wave field within the liquid. By adjusting the flow rate of the microfluidic pump, gas was injected into the water through a needle, forming a cavitation bubble at the antinode of the standing wave. Under the influence of the acoustic radiation force, the bubble was automatically trapped and held stably near the antinode within the standing wave field. By varying the amplitude or frequency of the sound pressure, the cavitation bubble could be induced to transition from spherical to nonspherical oscillations. The nonspherical oscillation of the cavitation bubble generated micrometer-scale acoustic streaming in the vicinity of the bubble. This experimental technique closely resembles those described in Refs. [2], [24], and [25].

To capture the dynamic behavior of cavitation bubbles, an LED lighting system with adjustable light intensity illuminated the bubbles. A high-speed camera system enabled clear visualization of the bubble outlines on a computer screen. The deformation evolution of the bubble from spherical to nonspherical oscillations was captured by a high-speed camera. The high-speed camera was set to a resolution of 768 × 512 pixels, a frame rate of 33,000 fps, and an exposure time of 5 µs.

To observe acoustic microstreaming using stable nonspherical oscillating bubbles, a continuous laser illumination system was essential. The high-speed camera was configured with a resolution of 960 × 768 pixels, frame rate of 500 fps, and exposure time of 1000 µs. After capturing the real-time images, we superimposed them frame-by-frame using ImageJ software, allowing us to obtain the acoustic microstreaming image induced by the oscillation of non-spherical bubbles. Acoustic microstreaming is represented by the trajectories of tracer particles. Consequently, prior to bubble injection, we added approximately 0.04 ml of solution containing red fluorescent tracer particles with a diameter of 800 nm to degassed deionized water and mixed the solution thoroughly. Subsequently, we deactivated the LED lighting system and activated the continuous laser lighting system, which featured adjustable intensity. We then adjusted the distance between the biconcave and cylindrical plano-convex lenses to create a plane light source within the liquid. This plane light illuminated the cavitation bubbles and surrounding tracer particles. Finally, the movement trajectories of the particles were captured using the high-speed camera.

### 2.2 Finite element numerical calculation model

**Fig. 2:**
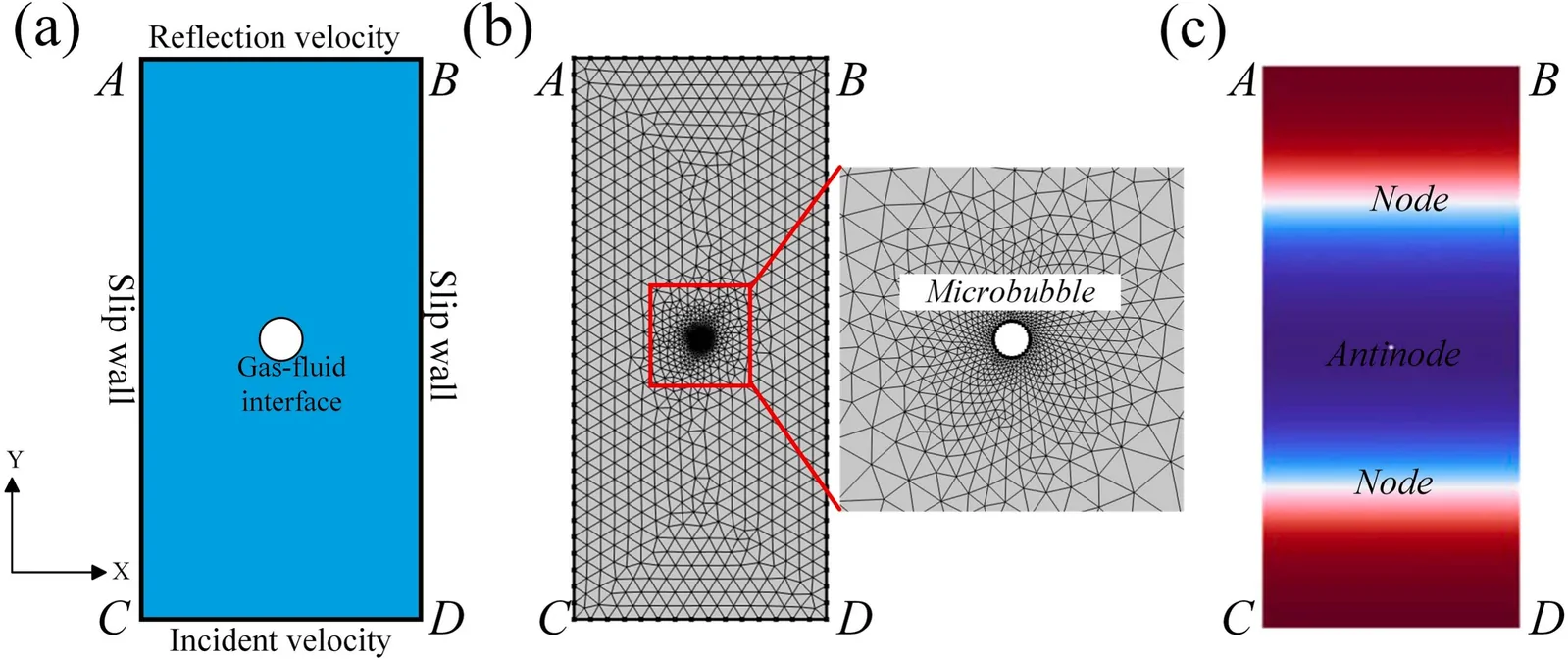
(a) Schematic diagram of physical geometric model; (b) Numerical geometric model grid division diagram; (c) Sound pressure distribution diagram.

A numerical calculation model for the nonspherical oscillation of a single bubble in an ultrasonic standing wave field was developed using the Computational Fluid Dynamics (CFD) module and arbitrary Lagrangian-Eulerian (ALE) method in COMSOL Multiphysics software. Fig. 2(a) illustrates the schematic of the standing wave field in pure water, where the blue region denotes the pure water domain and the white region indicates the presence of bubbles. In the computational domain, wall CD serves as the boundary condition for the incident traveling-wave velocity, whereas wall AB functions as the boundary condition for the reflected traveling-wave velocity. The superposition of the incident and reflected traveling waves results in the formation of a standing wave field within the computational domain of the model. To comprehensively examine the non-spherical oscillations and acoustic microstreaming of bubbles in this standing wave field, the heights of walls AC and BD are set at 12 cm. The initial bubble is positioned at the midpoint between the X and Y coordinates, and its radius is denoted by *R*_0_. To prevent the walls from introducing spurious vorticity and viscous damping that would suppress non-spherical bubble deformation, walls AC and BD are configured as sliding walls (zero normal penetration, zero tangential shear). Additionally, walls AB and CD are placed at a sufficient distance of 5 cm from the bubble to ensure the computational boundaries do not perturb the flow field. The computational domain is discretized with a free triangular mesh. Given that the primary focus of the study is on the bubble deformation process rather than on the interior of the bubble, the bubble domain is excluded from the liquid domain using Boolean operations, retaining only the liquid computational domain. A moving mesh ALE module is employed to capture the changes on the bubble surface, with the computational liquid domain serving as the free deformation domain of the bubble. This allows the mesh to move freely, optimizing the bubble deformation shape while ensuring that the grid speed is aligned with the fluid speed. The mesh is configured for fluid dynamics using a predefined mesh-size set for refinement. To improve the accuracy of the bubble interface deformation, mesh division at the gas–liquid interface is established with a fixed element distribution of 80. The specific grid divisions are illustrated in Fig. 2(b).

This study examines the nonspherical oscillation of bubbles within a standing wave field and the induced acoustic microstreaming. Assuming that the fluid domain surrounding the bubble is incompressible, the mass [Eq. (1)] and momentum conservation equations [Eq. (2)] follow from [26] and are given by:

(1) $$\frac{\partial \rho }{\partial t}+\nabla \cdot \left(\rho u\right)=0$$

(2) $$\rho \frac{\partial u}{\partial t}+\rho u\cdot \nabla u=-\nabla p+\nabla \cdot \left(\mu \left(\nabla u+{\left(\nabla u\right)}^{\text{T}}\right)\right)+\rho g+F$$

(3) $$F=\sigma \kappa_{m}$$

where u denotes the fluid velocity, p indicates the fluid pressure, μ is the dynamic viscosity of the fluid, ***g*** signifies the acceleration due to gravity. ***F*** represents the surface tension exerted on the bubble surface, which acts at the gas–liquid interface. The parameter $\kappa_{m}$ denotes the average curvature of this interface, and its expression is $\kappa_{m}=-\nabla \cdot n$, ***n*** is the unit normal vector.

Fig. 2(c) shows that the sound field is characterized as a standing wave. Within the computational domain, the wave propagating in the positive Y-direction is designated as the incident traveling wave, and its vibration velocity is defined as [27]:

(4) $$v_{1}\left(t,y\right)=v_{0}\cos\left(\omega t-ky\right)$$

The wave propagating along the negative Y-axis is defined as the reflected traveling wave, and its vibration velocity is:

(5) $$v_{2}\left(t,y\right)=v_{0}\cos\left(\omega t+ky\right)$$

Based on the principle of wave superposition and by using trigonometric identities,

(6) $$v\left(t,y\right)=v_{1}\left(t,y\right)+v_{2}\left(t,y\right)=2v_{0}\cos\left(\omega t\right)\cos\left(ky\right)$$

(7) $$v_{0}=\frac{\mathrm{Pa}}{\rho c}$$

where the angular frequency is ω=2πf, and f refers to the frequency of the sound wave. The wave number is defined k=ω/c

Under isothermal conditions and in the absence of gas diffusion, the gas contained within the bubble can be approximated as an ideal gas, adhering to the ideal gas law. Consequently, the instantaneous pressure within the bubble is given by:

(8) $$p_{g}=p_{g0}{\left(\frac{V_{0}}{V_{i}}\right)}^{\gamma }$$

(9) $$p_{g0}=p_{0}+\frac{2\sigma }{R_{0}}$$

(10) $$V_{i}=\frac{4}{3}\pi R_{i}$$

where $R_{i}$ signifies the instantaneous radius. The equivalent radius $R_{i}$ is derived through the integration at the gas–liquid interface. At this interface, the continuous normal pressure equilibrium is maintained, and concurrently, the continuous normal velocity boundary condition [28] is satisfied, which is expressed as:

(11) $$n\cdot \left[-pI+\mu \left(\nabla u+{\left(\nabla \upsilon \right)}^{\text{T}}\right)\right]=-p_{g}\cdot n$$

(12) $$u_{\mathrm{mesh}}\cdot n=u_{\mathrm{fluid}}\cdot n$$

where $u_{\mathrm{mesh}}$ is the mesh velocity and $u_{\mathrm{fluid}}$ is the movement velocity of the bubble interface.

To analyze the shear stress effects induced by nonspherical bubble oscillations, the viscous shear stress is computed from the velocity field solution using COMSOL built-in variables. In the Equation View of the software, the built-in shear rate variable *sr* for the two-dimensional case is defined as:

(13) $$\mathrm{sr}=\sqrt{2(S_{\mathrm{xx}}^{2}+S_{\mathrm{xy}}^{2}+S_{\mathrm{yx}}^{2}+S_{\mathrm{yy}}^{2})}$$

where the strain-rate tensor components are $S_{\mathrm{xx}}=\frac{\partial u}{\partial x}$, $S_{\mathrm{xy}}=S_{\mathrm{yx}}=\frac{1}{2}\left(\frac{\partial u}{\partial y}+\frac{\partial v}{\partial x}\right)$, $S_{\mathrm{yy}}=\frac{\partial v}{\partial y}$, where *u* and *v* are the *x* and y-component of velocity, respectively. Multiplying the shear rate by the dynamic viscosity μ gives the equivalent scalar magnitude of the viscous shear stress *τ*:

(14) $$\tau =\mu \cdot sr=\mu \cdot \sqrt{2S:S}$$

This value is computed at each mesh node in the fluid domain and output as a contour map, representing the intensity of bulk viscous shearing at each spatial location. No stress balance conditions at the gas–liquid interface are involved.

The MUMPS direct solver is employed in the computational model because of its robustness. The calculation spans 15 periods with a time step of *T*/100 (*T* = 1/*f*) and a relative tolerance of 0.02. In addition, automatic mesh redivision is implemented. When the mesh quality falls below 0.2, the computational model will remesh the geometric mesh to address issues related to mesh distortion and fluid flow. Table 1 lists the physical parameters utilized in the computations.

**Table 1:** Physical variables incorporated into the computation.

Symbol	Definition (value)
ρ	Liquid density (10^3^ kg/m^3)^
μ	Liquid viscosity (0.001 Pa·s)
*p*	Pressure within the fluid
σ	Surface tension (0.073 N/m)
c	Velocity of sound (1480 m/s)
*P*_a_	Driving pressure amplitude
*v*_0_	Vibrational velocity for standing waves
*V*	Instantaneous volume of the bubble
*V*_0_	Initial volume of the bubble
γ	Polytropic index of the gas (1.4)
*k*	Wave number
*R*	Instantaneous microbubble radius
*R*_0_	Microbubble initial radius
*p*_g_	Microbubble’s internal pressure
*p*_g0_	Initial gas pressure inside the bubble
*p*_0_	Ambient pressure (1.013 × 10^5^ Pa)
*f*	Driving pressure frequency (24 kHz)
*S*	Strain-rate tensor

## 3 Results and discussion

### 3.1 Nonspherical oscillation characteristics of cavitation bubbles

**Fig. 3:**
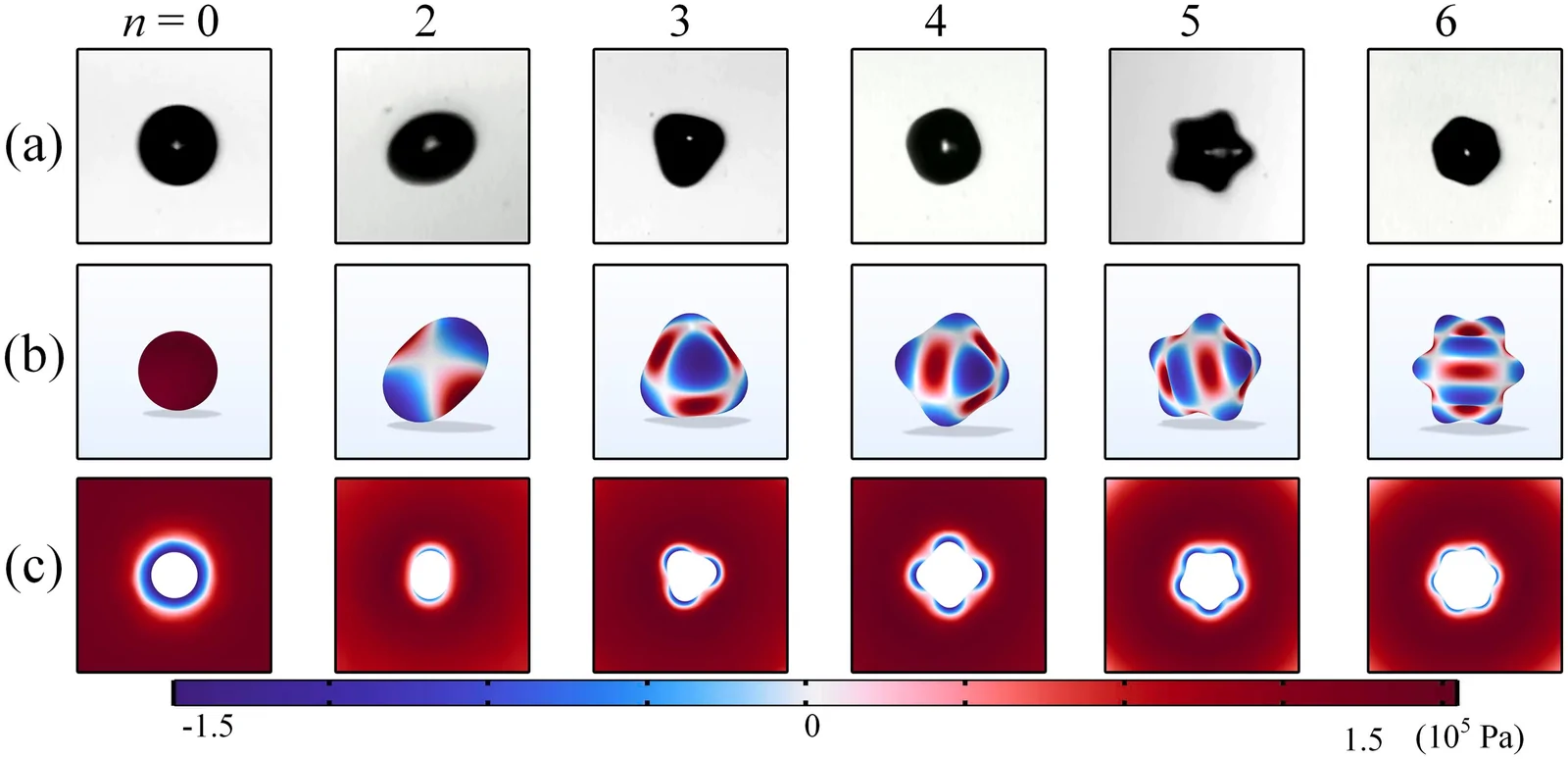
(a) Oscillation of bubbles in various shapes modes; (b) Three-dimensional oscillations corresponding to distinct bubble shape modes; (c) Pressure distribution associated with oscillation of different bubble shape modes.

A bubble within a standing-wave acoustic field can be regarded as a forced harmonic oscillator. Under specific ultrasonic driving conditions, bubbles undergo spherical expansion and compression near the pressure antinode of the acoustic wave. When the amplitude or frequency of the driving acoustic pressure deviates from specific values, the spherical interface becomes unstable. This causes the bubble to transition from spherical to non-spherical shape oscillations, or even leads to interface rupture. Fig. 3(a) presents the spherical oscillation morphology (*n* = 0) and the dominant non-spherical oscillation modes of the bubble captured by a high-speed camera, with non-spherical modes labeled by their mode numbers (*n* = 2, 3, 4, 5, 6, …). The profile of the bubble interface is described by the Legendre polynomial [22]:

(13) $$r_{s}\left(\theta ,t\right)=R_{0}+\sum_{n=0}^{N}a_{n}e^{-i\omega_{n}t}P_{n}\left(\cos\theta \right)$$

where *R*_0_ is the spherical radius of the bubble, $a_{n}$ signifies the deformation amplitude of the *n*^th^ mode, $\omega_{n}$ is the natural frequency of a given shape mode *n*, and *P_n_* is the Legendre polynomial of the *n*^th^ order with cosθ . The bubble-shape modes captured in the experiment were derived from two-dimensional planar projections using a high-speed camera. Bubbles oscillate in a three-dimensional, non-spherical manner in a free field. To account for this, we also performed numerical simulations of the three-dimensional oscillations. Fig. 3(b) presents the three-dimensional oscillation of each shape mode of the bubble as obtained from the numerical simulations, effectively illustrating the changes occurring on the bubble surface during oscillation in different shape modes. To investigate the factors contributing to bubble deformation, we developed a model utilizing the numerical calculation method described in Section 2.2 to examine the pressure distribution around the bubble surface. Fig. 3(c) shows the pressure cloud diagram of a bubble oscillating under the influence of ultrasonic waves. When a bubble exhibits spherical oscillation, a uniform pressure surrounds its surface. In contrast, when the bubble adopts various shape modes, the protruding regions at the bubble interface correspond to extreme pressure values. For example, when the bubble undergoes shape mode 4 oscillation, the four extreme-pressure values align with the four lobes surfaces of the bubble. This phenomenon arises from the interplay between the surface tension of the bubble and the pressure gradient acting on its surface.

For the experimentally observed bubbles exhibiting different shape-mode oscillations, parametric instability is the most relevant surface instability. A generic feature of parametric instability is that shape oscillations are most readily excited on the bubble surface when the driving frequency satisfies $\omega =2\omega_{n}$, where ω=2πf is the driving angular frequency and $\omega_{n}$ is the natural frequency of a given shape mode *n*. According to Lamb’s theory, the natural frequency $\omega_{n}$ of shape modes *n* of bubbles is given as [29]:

(14) $$\omega_{n}^{2}=\frac{(n-1)(n+1)(n+2)\sigma }{\rho R_{0}^{3}}$$

**Fig. 4:**
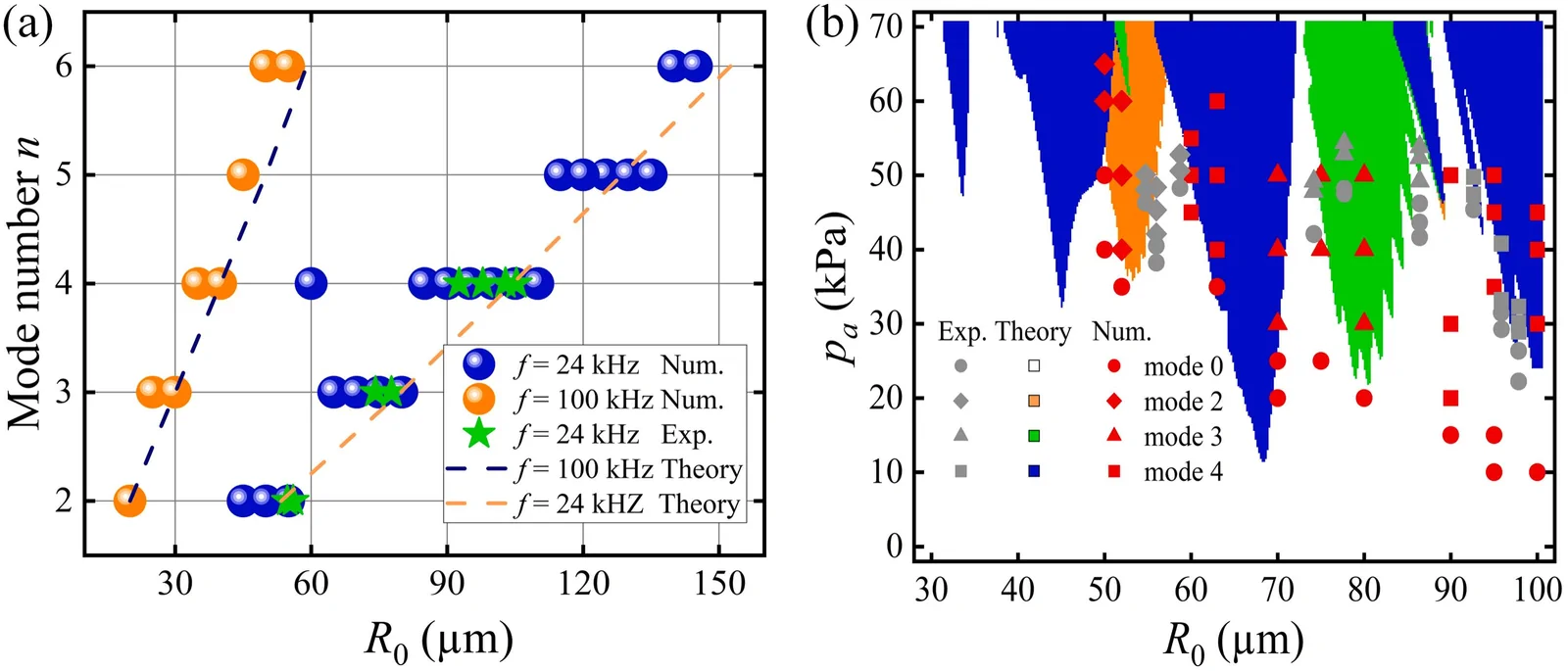
Shape mode oscillations of cavitation bubbles. (a) Dominant shape mode number *n* for different *R*_0_ at different driving frequencies (*f*)*.* Green stars denote experimental data under 24 kHz; Blue solid circles and orange solid circles represent numerical results under 24 kHz and 100 kHz, respectively. Dark blue and orange dashed curves correspond to Lamb's theoretical predictions for 100 kHz and 24 kHz, respectively. (b) Phase diagram of the *R*_0_–*p_a_* at *f* = 24 kHz. Solid circles, rhombuses, triangles, and squares represent the dominant shape modes (*n* = 0, 2, 3, and 4) observed in COMSOL numerical results, respectively. The gray symbols of corresponding shapes denote experimental observations. The color filled regions indicate the instability zones of each mode, similar to that in Ref. [31].

Eq. (14) provides a theoretical prediction of the dominant shape mode number *n* for a given *R*_0_ at different driving frequencies. The dominant shape mode numbers of bubbles with various *R*_0_ were computed using the COMSOL numerical model at driving frequencies of 24 kHz and 100 kHz under a sound pressure of 50 kPa, as shown in Fig. 4(a). The orange dashed curve represents Lamb's theoretical prediction from Eq. (14), green stars denote experimental data at 24 kHz, and blue and orange circles correspond to numerical results at 24 kHz and 100 kHz, respectively. The numerical results at both frequencies agree well with the Lamb expression and experimental data, confirming the validity of the numerical model. The results indicate that under constant sound pressure, the dominant shape mode number increases with increasing initial bubble radius at a fixed driving frequency or with decreasing driving frequency at a fixed *R*_0_. In other words, larger bubbles at lower frequencies are more susceptible to high-order nonspherical oscillations, consistent with experimental findings reported in Refs. [22], [23], and [30].

Fig. 4(b) shows the *R*_0_–*p*_*a*_ phase diagram at *f* = 24 kHz, including experimental data, numerical results, and the theoretical instability regions from Ref. [31], with good agreement among the three. The results demonstrate that once the pressure threshold for shape oscillations is exceeded, further increases in sound pressure do not alter the dominant shape mode number, which depends solely on *R*_0_. This is consistent with the conclusion of Versluis et al. [2].

### 3.2 Evolution of oscillation of cavitation bubbles in different shape modes

**Fig. 5:**
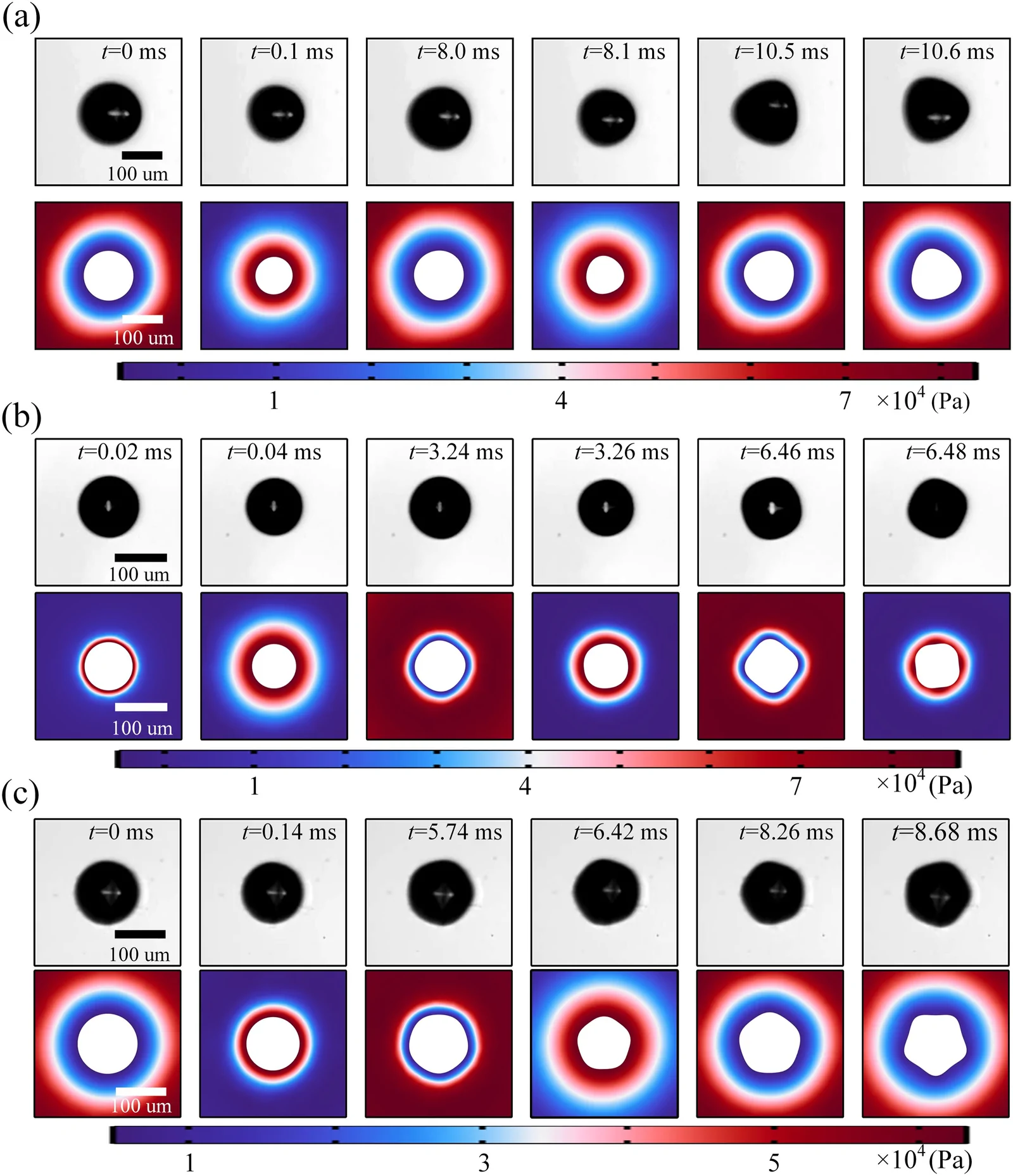
Experimental and numerical simulations illustrating the time evolution of bubbles transitioning from spherical oscillation to oscillations in various shape modes: (a) Oscillation of bubble shape mode 3 (*n* = 3); (b) Oscillation of bubble shape mode 4 (*n* = 4); (c) Oscillation of bubble shape mode 5 (*n* = 5).

To analyze the evolution of surface deformation in cavitation bubbles, we employed a high-speed camera to capture their progression from spherical to shape oscillations of modes 3, 4, and 5. Fig. 5(a) illustrates that at a driving frequency of *f* = 24 kHz, with driving sound pressure ranging from *p_a_* = 0.793 to 0.806 atm and initial radius *R*_0_ of the bubble measured at 77.68 µm based on calibration scale parameters, the bubble transitions from spherical to shape mode 3 oscillation. During the time interval *t* = 0–0.1 ms, the bubble retains its spherical shape while undergoing compression. Correspondingly, numerical simulations indicate alternating changes in the surrounding pressure, reflecting the compression and expansion dynamics of the bubble. In the time frame *t* = 8.0–8.1 ms, minor triangular deformations are observed at the bubble surface. Experimental observations confirm that the bubbles are also compressed during similar deformations and flipping. Numerical simulations of surrounding sound pressure corroborate these findings. During *t* = 10.5–10.6 ms, the bubbles display pronounced triangular deformation and undergo flipping. The bubbles elongate in the horizontal direction, accompanied by local depressions, which is also evident in the numerical simulations.

Fig. 5(b) illustrates the evolution of the bubble from volume pulsation to shape mode 4 oscillation at a frequency of *f* = 24 kHz and applied sound pressure of *p_a_* = 0.921–0.945 atm. The initial radius of the bubble, measured experimentally, is 87.52 µm. Both experimental and numerical simulations were performed. The bubble interface in the experiment exhibited a distinct four-lobed structure. During the oscillation period, specifically between *t* = 0.02–0.04 ms, the high-speed camera recorded the volume pulsation trend of the bubbles, which retained a spherical shape. During *t* = 3.24–3.26 ms, the bubble ceased to maintain its spherical form during volume pulsation and began to exhibit interface deformation resembling a quadrilateral. During *t* = 6.46–6.48 ms, the four symmetrical lobes bodies underwent regular protrusion and contraction, resembling a rotating square outline (see Supplementary Video S1 and S2). As the experiment represents a two-dimensional projection, subtle pattern rotations were observed, suggesting the potential presence of initial angular disturbances.

Fig. 5(c) presents the time-series diagrams of the bubble, illustrating the transition from spherical oscillations to shape mode 5 oscillation within a standing wave field at a constant frequency. This occurs when the applied driving sound pressure ranges from 0.388–0.491 atm, with an initial bubble radius of *R*_0_ = 87.63 µm, as determined through both experimental and numerical simulations. During the interval *t* = 0–0.14 ms, frames captured by a high-speed camera reveal the volume pulsation of the bubbles. When the amplitude of the applied sound pressure surpasses a specific threshold, the bubble exhibits pentagonal oscillation between *t* = 5.74–6.72 ms, characterized by a notable symmetry in its oscillation. From *t* = 8.26 to 8.68 ms, the bubble consistently demonstrates fifth-order shape oscillation.

### 3.3 Oscillatory flow and acoustic microstreaming induced by nonspherical bubble oscillation

**Fig. 6:**
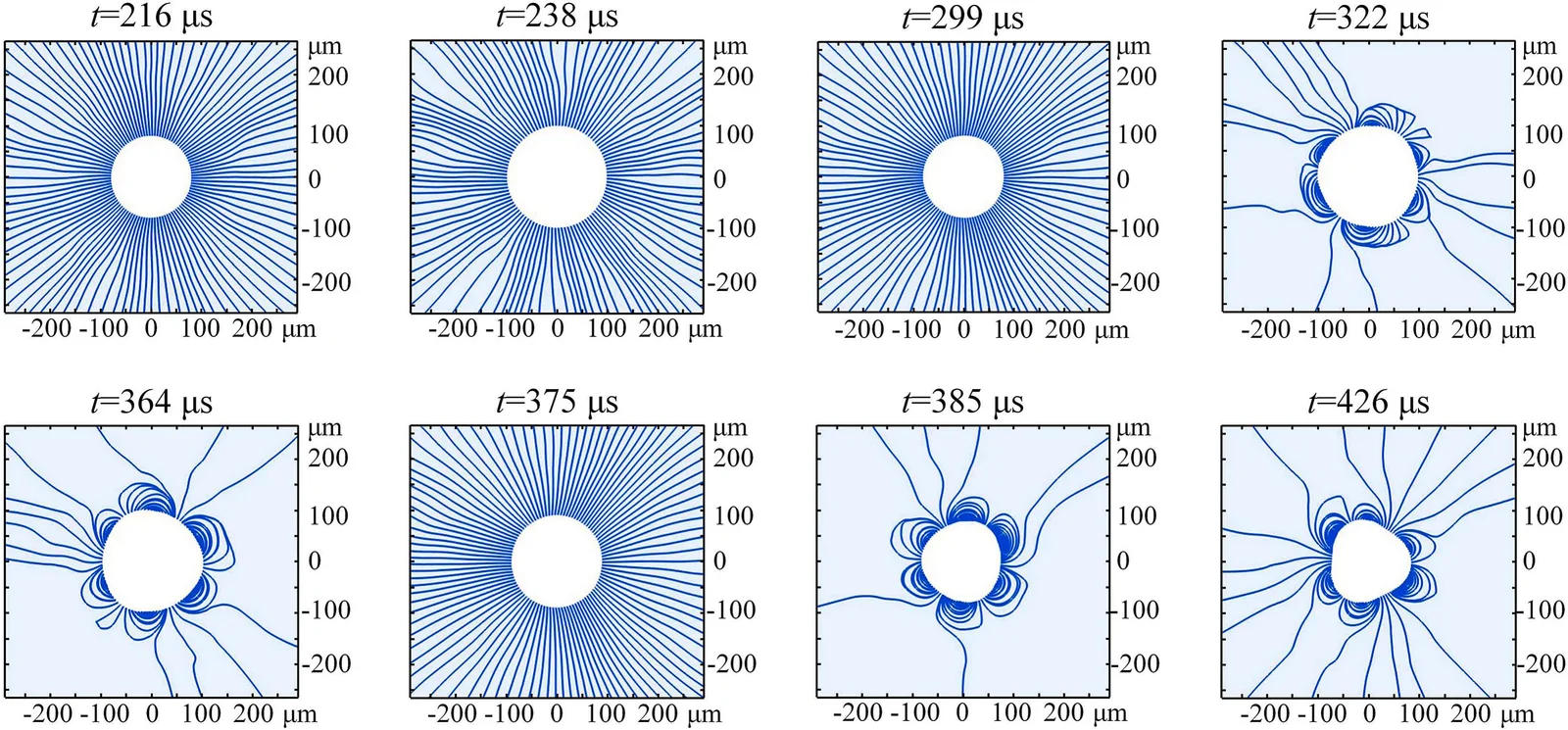
Temporal and spatial evolution of the flow field generated during the transition of the bubble from spherical to shape mode 3 oscillation.

Fig. 6 illustrates the oscillatory flow spatial evolution induced by a bubble oscillating at *f* = 24 kHz under an acoustic pressure of *p_a_* = 30 kPa with an initial radius *R*_0_ = 75 μm, where the bubble is exposed to ultrasonic waves directed along the y axis. During *t* = 216–238 μs, the bubble oscillates in a spherical mode and the surrounding liquid flows radially to form luminous streamlines. At *t* = 299–322 μs, the bubble maintains spherical oscillation but as it reaches maximum amplitude, slight interfacial deformation occurs and six small vortex structures appear in the adjacent liquid; the closure of the velocity streamline in the upper-right corner likely originates from this minor deformation, which induces a locally weakened flow. In the interval *t* = 364–375 μs, the bubble interface reverts from a shape mode 3 configuration to a spherical shape, with the surrounding streaming lines transitioning from six vortices back to luminous streamlines. At *t* = 385 μs, the interface exhibits a phase-reversed shape mode 3 oscillation, with six equally sized vortex structures appearing around the bubble, and by *t* = 426 μs, a stable phase-reversed shape mode 3 oscillation is established, again accompanied by six vortices around the bubble. These transient spatiotemporal dynamics, spanning from spherical oscillation to shape mode 3, demonstrate that the six-vortex microstreaming field arises from axisymmetric nonspherical oscillations at the bubble interface. The numerical results further indicate that when a bubble undergoes coupled spherical and shape mode 3 oscillation, the number of surrounding vortex structures equals twice the shape mode number *n*, which is consistent with the findings of Doinikov et al. [16].

**Fig. 7:**
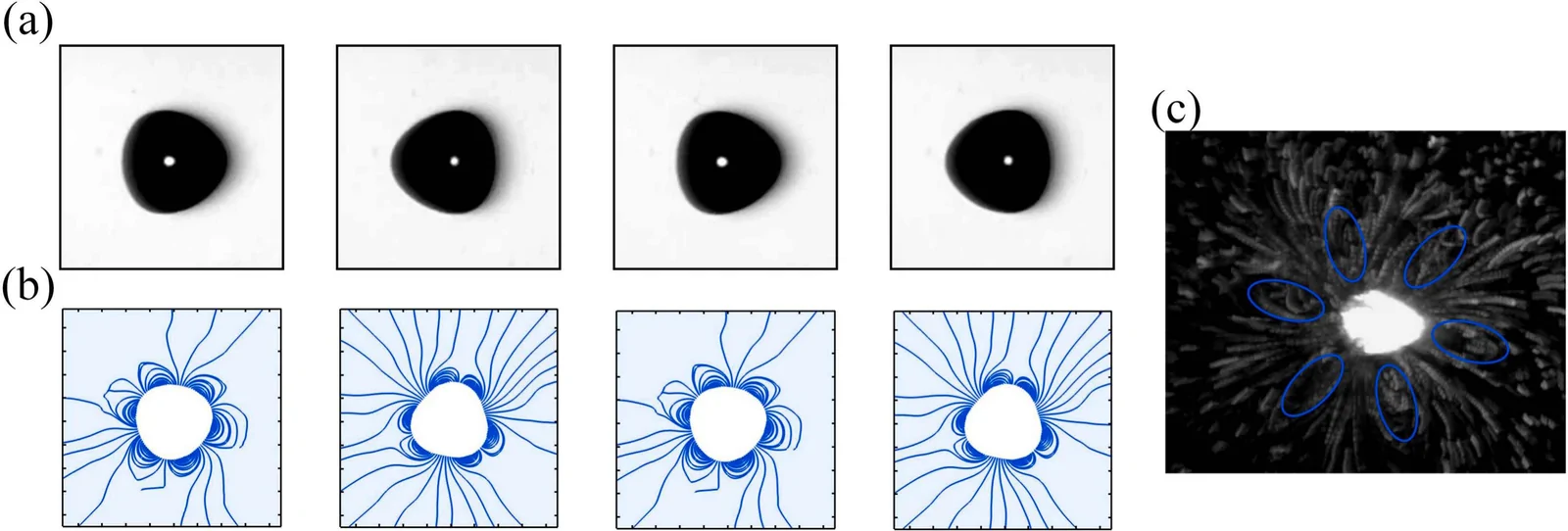
(a) Stable oscillation deformation of bubble shape mode 3; (b) Instantaneous streamline snapshots from numerical simulation, corresponding to the phases in Fig. 7. (a); (c) Experimental image of acoustic microstreaming induced by stable oscillation of bubble shape mode 3.

Fig. 7 illustrates the transient oscillatory flow and time-averaged acoustic microstreaming induced by stable oscillations of bubble shape mode 3 at a driving frequency of *f* = 24 kHz. The experimental sound pressure amplitude was *p_a_* = 52.46 kPa while the numerical simulation was conducted at *p_a_* = 45 kPa, with an initial bubble radius of *R*_0_ = 78.26 μm. Under the parametric resonance condition, the oscillation frequency of bubble shape mode 3 is *f*_3_ = *f* /2 ≈ 12 kHz yielding a shape mode 3 oscillation period *T*_3_ = 2*T* equal to twice the acoustic period. Consequently, a π phase reversal of the bubble interface occurs every acoustic period *T*, manifesting as alternating left-protruding and right-protruding configurations in the projection view. Fig. 7(a) shows four representative frames spanning three acoustic periods of stable shape mode 3 oscillation. It can be observed that, during shape mode 3 oscillation, the alternating deformation of the bubble interface exhibits a phase difference of π/2 relative to the spherical oscillation (see Supplementary Video S3). Fig. 7(b) presents the corresponding instantaneous streamline snapshots of the velocity field at the phases illustrated in Fig. 7(a). These instantaneous streamlines represent the first-order oscillatory flow, which is inherently tied to the instantaneous bubble interface configuration. Consequently, the vortex structure shown in Fig. 7(b) is asymmetric and varies with the bubble deformation at each instant. Nevertheless, this periodic oscillatory flow drives acoustic microstreaming through Reynolds stress accumulation over each acoustic period. Fig. 7(c) shows the experimental acoustic microstreaming pattern obtained by time-averaging sequential video frames using ImageJ software. This second-order steady flow is visualized by superimposing particle trajectory images captured by a high-speed camera over multiple acoustic periods (see Supplementary Video S4). The resulting six-vortex pattern exhibits perfect symmetry and, unlike the instantaneous flow, is independent of the instantaneous interface configuration. This symmetry emerges as a cumulative consequence of stable shape mode 3 oscillations sustained over many cycles.

**Fig. 8:**
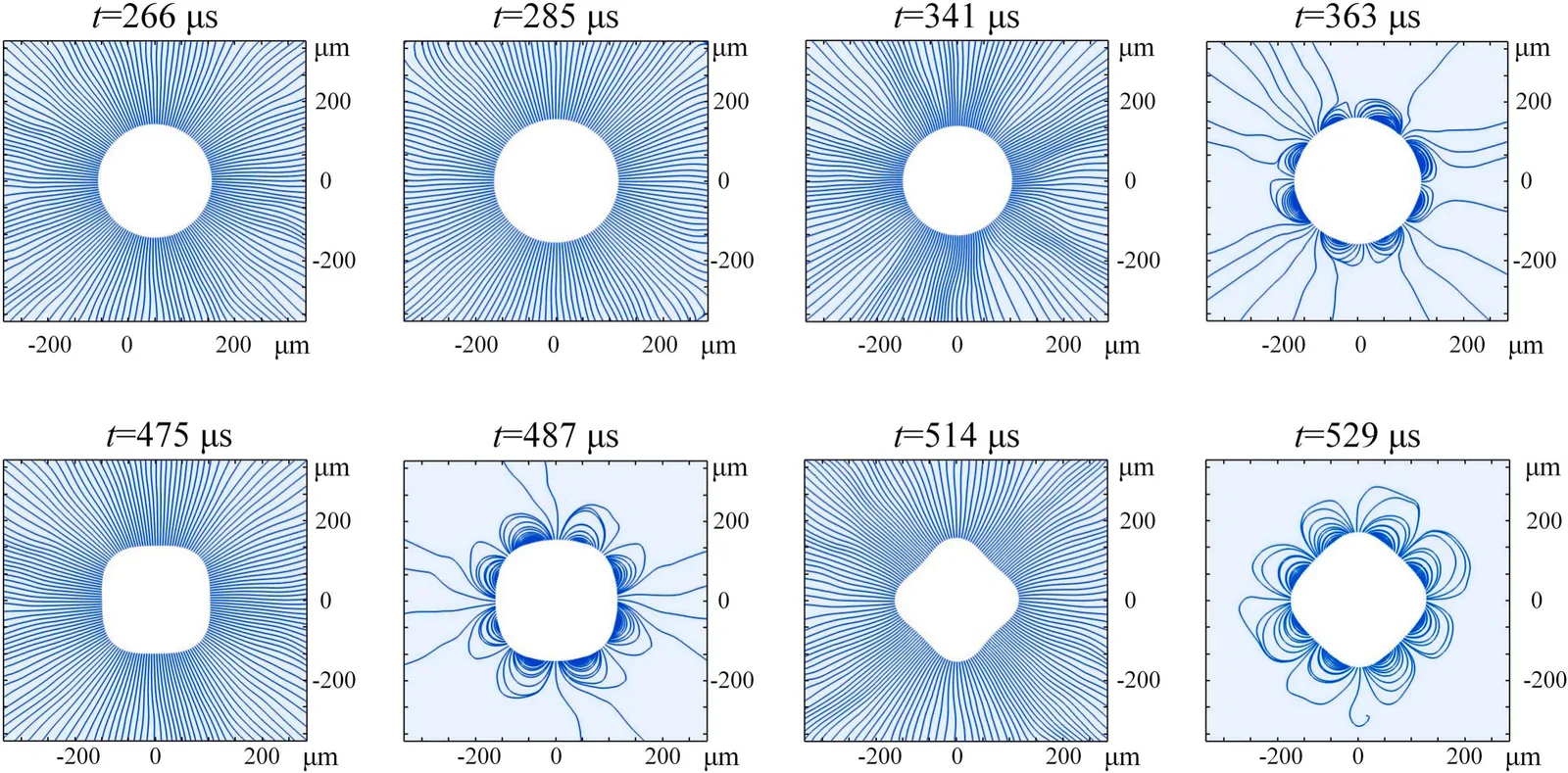
Temporal and spatial evolution of the flow field generated during the transition of the bubble from spherical to shape mode 4 oscillation.

Fig. 8 shows the temporal and spatial evolution of the flow field generated as a bubble transition from spherical to shape mode 4 oscillation, under the condition *p_a_* = 40 kPa, *f* = 24 kHz, and an initial bubble radius *R*_0_ = 95 µm. During the time interval *t* = 266–285 µs, the bubble undergoes spherical oscillation, and the velocity streamlines of the surrounding liquid display a radiant pattern. At *t* = 341 µs, the bubble interface begins to deform slightly. The absence of streamlines around the bubble is attributed to the ongoing expansion phase, during which the spherical oscillation mode governs the streamline characteristics in the bubble's vicinity. At *t* = 363 µs, when the bubble attains its maximum expansion, the interface adopts a distinct quadrilateral shape, accompanied by the emergence of eight symmetrical vortex structures surrounding the bubble. This observation suggests that the coupling between the spherical oscillation and the shape mode 4 oscillation influences the bubble dynamics. Notably, the vortex structure at maximum expansion is predominantly characterized by a fourth-order mode, a feature not previously observed in experiments. During *t* = 475–487 µs, the bubble interface again exhibits a well-defined quadrilateral shape. The bubble retains this quadrilateral geometry while expanding, and the surrounding liquid flow displays a radiant streamline pattern. At maximum expansion, eight larger vortex structures appear around the interface. Over the subsequent period of *t* = 514–529 µs, the bubble enters a state of periodic stable oscillation, while still exhibiting coupled oscillations of the spherical and shape mode 4 oscillation. Concurrently, an even larger vortex structure forms around the bubble interface. The following discussion examines the relationship between numerical simulations of the transient oscillatory flow and the time-averaged acoustic microstreaming observed experimentally during shape mode 4 oscillation of the bubble.

**Fig. 9:**
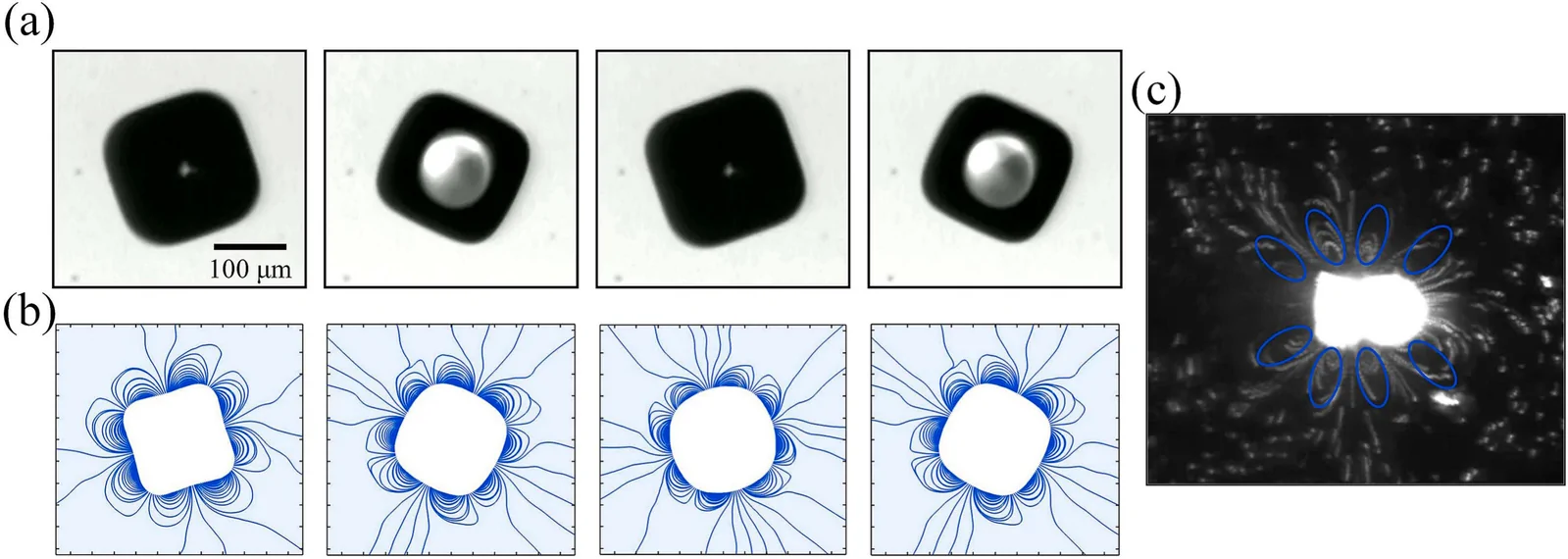
(a) Diagram of interfacial deformation during stable shape mode 4 oscillation of a bubble; (b) Corresponding instantaneous streamline snapshots of the velocity field at the phases illustrated in Fig. 9. (a); (c) Experimental image of acoustic microstreaming induced by stable oscillation of bubble shape mode 4.

Fig. 9 shows the transient oscillatory flow and time-averaged acoustic microstreaming induced by stable shape mode 4 oscillation at *f* = 24 kHz. The experimental and numerical sound pressure amplitudes were *p_a_* = 39.67 kPa and *p_a_* = 35 kPa, respectively, with an initial bubble radius of *R*_0_ = 98.32 μm. Under the parametric resonance condition, the oscillation frequency of shape mode 4 is *f*_4_ ≈ 13 kHz ≈ *f* / 2, falling within the parametric instability bandwidth, with its oscillation period *T*_4_ = 2*T*. A *π* phase reversal of the bubble interface therefore occurs every acoustic period *T*, manifesting as a 90° rotational alternation in the projected view. Fig. 9(a) shows four representative frames spanning three acoustic periods of stable shape mode 4 oscillation. It can be observed that during shape mode 4 oscillation, the alternating deformation of the bubble interface exhibits a phase difference of *π*/4 relative to the spherical oscillation (see Supplementary Video S5). The instantaneous streamlines in Fig. 9(b) and the time-averaged microstreaming in Fig. 9(c) follow the same physical mechanism as described for Fig. 7. The resulting pattern exhibits an eight-vortex structure with perfect symmetry, reflecting the cumulative consequence of stable shape mode 4 oscillations sustained over many cycles (see Supplementary Video S6).

### 3.4 Oscillatory flow velocity distribution during the transition from spherical to nonspherical oscillation

**Fig. 10:**
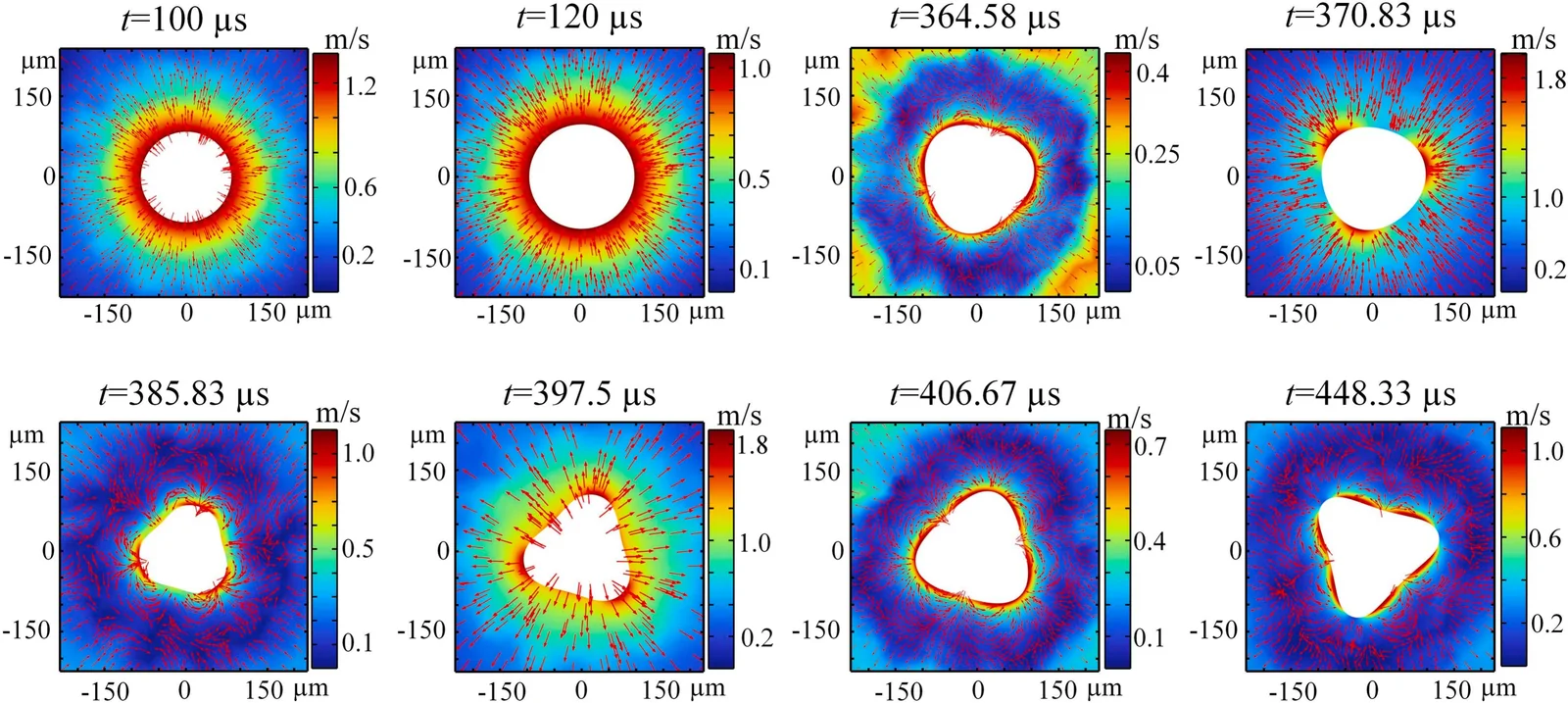
Evolution of the instantaneous velocity distribution of a bubble during the transition from spherical to shape mode 3 oscillation.

Fig. 10 shows the instantaneous velocity distribution and velocity vectors of a bubble transitioning from spherical to shape mode 3 oscillations, at *f* = 24 kHz, *p_a_* = 0.18 MPa and *R*_0_ = 80 µm. At *t* = 364.58 µs, the bubble undergoes triangular deformation near its maximum expansion; the axisymmetric flow field reorganizes to reflect the shape mode 3 oscillation, the radial velocity distribution becomes non-uniform, and six symmetric vortices appear around the bubble surface, exhibiting an anti-fountain-like instantaneous flow pattern (arrows indicate flow direction). At *t* = 370.83 µs, the bubble enters compression while maintaining the *n* = 3 shape mode, with arrows uniformly distributed around it. At *t* = 385.83 µs, the bubble reaches its minimum compression, where six vortices reappear near the interface. Compared with the anti-fountain-like pattern at maximum expansion, the instantaneous arrow directions at this stage are reversed. The arrows originate from the top of the bubble and return to the equator along a closed loop, forming a fountain-like pattern. At *t* = 397.5 µs, the *n* = 3 mode reappears during expansion, again with uniformly distributed arrows. From *t* = 406.67 µs to 448.33 µs, the bubble enters a stable reverse oscillation phase, during which asymmetric structures emerge around it. The anti-fountain-like and fountain-like instantaneous flow patterns observed at *t* = 364.58 µs and 385.83 µs are consistent with the time-averaged microstreaming patterns reported in Refs. [20], [32], [33], suggesting that these instantaneous oscillatory structures underlie the steady streaming behavior. These oscillatory flow patterns arise from the interaction between radial and axisymmetric shape mode oscillations.

### 3.5 Shear stress characteristics of acoustic microstreaming induced by nonspherical oscillation of a cavitation bubble

**Fig. 11:**
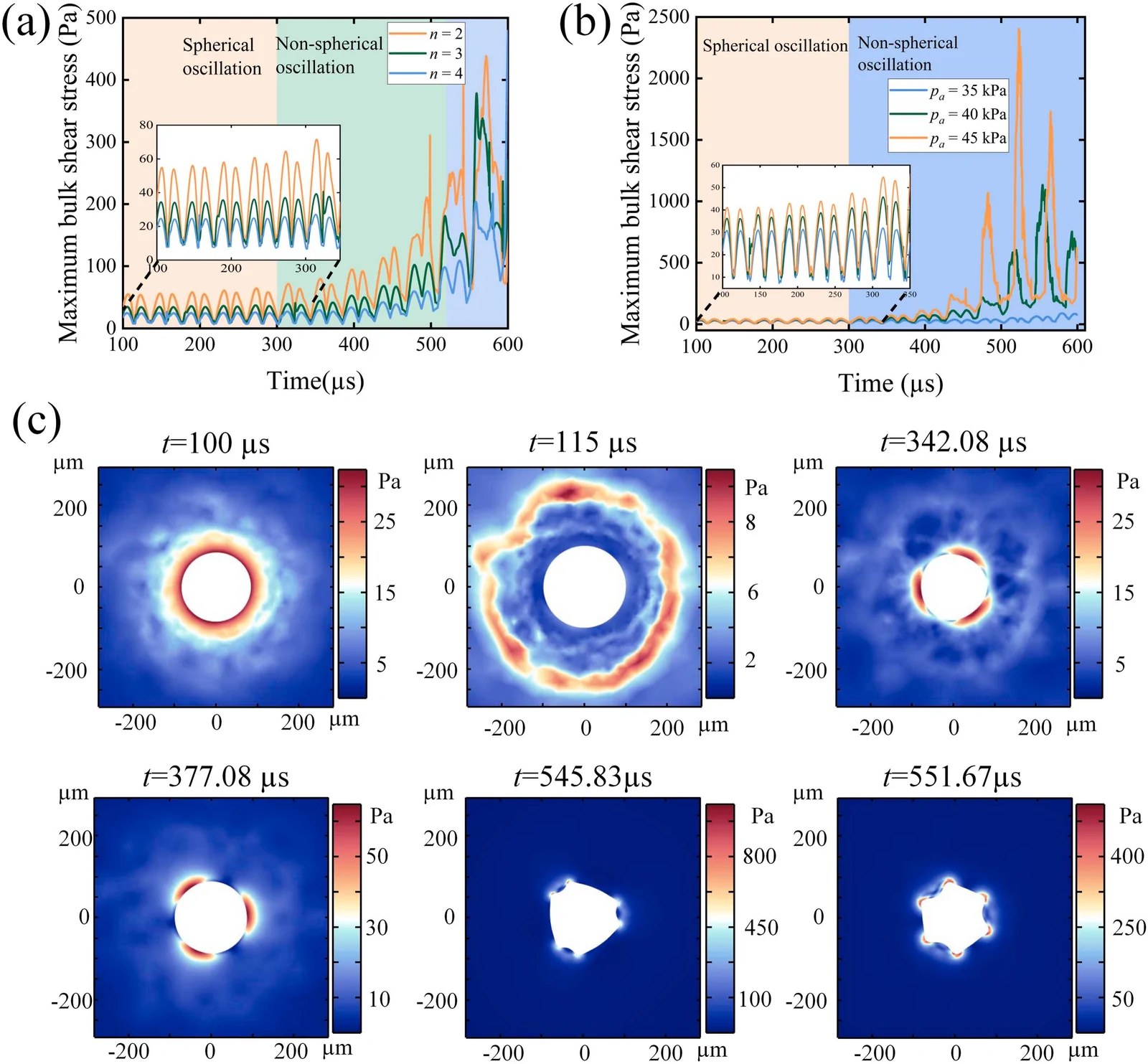
(a) Effect of shear stress on the transition of bubbles from spherical oscillation to various shape modes, under constant sound pressure and frequency; (b) Shear stress produced during the transition of bubbles from spherical to shape mode 3 oscillations under different sound pressure amplitude while maintaining constant frequency and bubble radius; (c) Temporal evolution diagram illustrating the spatial distribution of shear stress resulting from the transition of bubbles from spherical to nonspherical oscillation.

The nonspherical oscillation of cavitation bubbles induces an uneven distribution of the flow field surrounding the bubbles, leading to variations in the shear stresses in their vicinity. This phenomenon has potential value in biomedical applications, including drug delivery. To explore this further, we calculated and analyzed the changes in the maximum shear stress around the bubbles as they oscillated from spherical to nonspherical shapes. The shear stress τ was evaluated using the COMSOL built-in expression τ=μ·sr , the dynamic viscosity multiplied by the shear rate, which yields the equivalent scalar magnitude of the viscous stress tensor and represents the intensity of bulk viscous shearing at each point in the fluid. Fig. 11(a) illustrates the temporal evolution of maximum shear stress surrounding the bubble for various shape modes (*n* = 2, 3, 4) at *p_a_* = 35 kPa and *f* = 24 kHz. During the spherical oscillation phase (indicated by the orange section in the figure) and the weak nonspherical oscillation phase (represented by the green section), the amplitude of the maximum shear stress generated during the transition from volume spherical to the bubble shape mode 2 oscillation is markedly greater than that in the higher-order shape mode 3 or 4. A likely explanation is that *n* = 2 represents the lowest-order deformation mode for a bubble to deviate from a spherical shape, possessing the lowest energy excitation threshold. During the initial phase of volume pulsation, a significant surface velocity gradient can arise from symmetry deformation. In contrast, *n* = 3 and *n* = 4 correspond to higher-order asymmetric deformations that require a greater energy input for excitation. At the onset of oscillation, the amplitude of deformation is smaller and the velocity gradient is less pronounced, leading to reduced shear stress. As the bubble transitions into a strong nonspherical oscillation phase (indicated by the blue region), the higher-order modes are fully activated, enhancing the spatial gradient effect. Consequently, the amplitude of the maximum shear stress increases rapidly, surpassing that of the shape mode 2. This characteristic is also evident in the maximum shear stress distribution around the bubble, as shown in Fig. 11(c). Fig. 11(b) illustrates the impact of varying sound pressures (*p_a_* = 35, 40, 45 kPa) on the shear stress produced by the non-spherical oscillation of the bubble at driving frequency of *f* = 24 kHz and initial bubble radius of *R*_0_ = 75 µm. As the sound pressure increases, the amplitude of the maximum shear stress oscillation also increases, as observed in both the spherical (indicated by the orange section) and non-spherical oscillation regions (represented by the blue section). Additionally, higher sound pressures facilitate an earlier transition of the bubble from spherical to non-spherical oscillations. This phenomenon may be attributed to the enhanced energy input provided by the increased sound pressure, which accelerates the nonlinear deformation of the bubbles and subsequently intensifies the shear effect of the acoustic microstreaming. Fig. 11(c) also illustrates the evolution of the spatial distribution of shear stress as the initial bubble radius *R*_0_ = 75 µm transitions from spherical oscillation to non-spherical oscillation, under driving conditions of frequency *f* = 24 kHz and *p_a_* = 35 kPa. During the *t* = 100–115 µs interval, at the onset of spherical oscillation of the bubble, maximum shear stress is distributed in the axisymmetric ring pattern, with concentration near the equatorial plane of bubble. In the *t* = 342.08–377.08 µs interval, the bubble transitions into a phase of weak non-spherical deformation, during which the stress distribution exhibits fluctuations and gradually loses its axial symmetry. Upon entering the strong non-spherical oscillation phase in the interval *t* = 545.83–551.67 µs, the shear stress reveals eccentric distribution characterized by a multipolar mode structure. The emergence of localized high-stress regions directly correlates with areas of intense shear action within the acoustic microstreaming.

## 4 Conclusions

This study investigated the transition of a bubble from spherical to nonspherical oscillations in a standing-wave acoustic field using both experimental observation and numerical simulation. The results show that the nonspherical oscillation mode of the bubble is jointly governed by the driving frequency, the initial radius, and the acoustic pressure. The numerical simulation further reproduced the dynamic evolution of the flow field surrounding the bubble during this transition. When the bubble undergoes coupled oscillations involving the breathing mode (*n* = 0) and a shape mode (*n* ≥ 2), the number of vortices around the interface is twice the order of the shape mode. During the compression phase, the interfacial flow field exhibits a fountain-like pattern, while during the expansion phase, an anti-fountain-like pattern appears. The local shear stress induced by nonspherical oscillations is nonuniformly distributed, with its magnitude and spatial structure significantly influenced by the oscillation mode and the acoustic pressure. These findings deepen the understanding of the vortex formation mechanism induced by nonspherical oscillations of a free bubble, and provide a reference for the application of microbubbles in microfluidics and biomedicine.

## CRediT authorship contribution statement

**Zhongyang Wu:** Writing – original draft, Software, Formal analysis. **Jinfu Liang:** Writing – review & editing. **Chang Gao:** Visualization, Validation.

## Declaration of competing interest

The authors declare that they have no known competing financial interests or personal relationships that could have appeared to influence the work reported in this paper.
